# Identification of Health Characteristics of People with Physical Disability by Applying the PRECEDE Model

**DOI:** 10.3390/ijerph192215081

**Published:** 2022-11-16

**Authors:** Youngin Won

**Affiliations:** Department of Football Management, Munkyung College, Munkyung 36930, Republic of Korea; 4684645@naver.com; Tel.: +82-10-5011-5997

**Keywords:** physical disability, quality of life, health factors, Korea, PRECEDE model

## Abstract

This study aimed to diagnose the health characteristics of people with grade 1–4 physical disability (but without intellectual disability) by analyzing factors affecting their health through social, epidemiological, behavioral, and ecological diagnoses by partially applying the PRECEDE model. Those registered with physical disability in 2022 and attending a welfare center were selected, with samples extracted from Seoul, Gyeonggi-do, Chungcheong-do, Jeolla-do, and Gyeongsang-do. A total of 1200 people were selected, and the data of 1000 people were finally analyzed. A frequency analysis was performed to identify the participants’ characteristics. An independent *t*-test and one-way analysis of variance were performed to verify the hypotheses. To clarify the relationship between each variable, normality verification, confirmatory factor analysis, and structural equation model analysis were performed. First, the differences in factors influencing health promotion according to personal background variables (gender, age, and income level), including quality of life, showed partial differences according to age and income level. Second, according to disability-related variables (time of onset and disability grade), quality of life and health status showed partial differences. These results can be used as basic data or indicators to build a health promotion system that considers the health characteristics of individuals with a physical disability.

## 1. Introduction

The number of people with disability is continuously rising due to the aging population and the increase in risk factors for disability, such as accidents and diseases. Further, the population of those with a disability has increased from approximately 1.3 million in 2000 to 2.5 million in 2017, demonstrating an increase of 1,240,844 since 2000 [1]. Therefore, a paradigm shift is occurring toward supporting independent lives for people with disability and an increasing social interest in their welfare. As there are different grades and categories of disability, it is important to develop welfare policies that consider different characteristics to meet the various needs of people with disability. Recently, the South Korean government has implemented policies in response to the welfare needs of people with disability in various domains, such as life cycle, health, and education [1].

According to the 2017 Ministry of Health and Welfare survey on the needs of people with disability, medical and healthcare needs were the highest at 33.6%, followed by income security (40.0%) [1]. In other words, people with disability are highly interested in achieving economic stability and stability of physical health. However, according to statistics on disability and health reported by the National Rehabilitation Center in 2018, the average life expectancy of people with a physical disability is 74.4 years [2], which is lower than the average life expectancy of 82.4 years for South Koreans who are not disabled [3]. This can be examined in relation to the poor health status of people with a physical disability, who experience more health problems than those without a disability and are more susceptible to various complications, secondary disability, and chronic diseases [4]. Although the physical limitations of people with physical disability impose high demands on specialized rehabilitation hospitals, only a few of these hospitals exist. The healthcare system tailored to people without disability causes difficulties for those with a disability, such as long waiting times for treatment. Most hospitals for people with disability are located in metropolitan areas or large cities, rendering them difficult to access and contributing to the limited use of healthcare services [5].

To solve these social problems, various domestic policies are being created to address the difficulties faced by people with disability. However, several problems persist. First, welfare and health-related services for people with disability have not been able to satisfy their needs [5]. The health services implemented thus far for people with disability are either applied or provided in an integrated way that does not consider the individual characteristics of people with disability, such as age and gender, thus failing to meet their health service needs. Further, in a medical welfare service satisfaction survey for people with disability, only 14.4% answered “very satisfied”, while most expressed their dissatisfaction with the related services [1]. The Korea Disabled People’s Development Institute [5] reported that the effectiveness of the health promotion system for people with a physical disability was reduced. This was because the services were provided without acknowledging the differences in health status or health-related factors between people with disability, the participants of the system, and those without disability. Therefore, there is an urgent need for research on health services that consider the characteristics of people with disability to satisfy their health service needs.

Second, previous studies have revealed problems regarding the theoretical basis and indiscriminate application of services. Programs were developed for people without disability to support people with disability without considering their specific situations. People with a disability have the same need for a healthy life as those without disability. They deserve medical and health programs that can satisfy their needs without discrimination and as members of the local community. However, various studies [6,7,8,9] have revealed that programs considering the characteristics of people with disability, classified as the vulnerable group, have not been implemented despite these social problems. Therefore, a study that considers all parties’ interests is needed to analyze the characteristics of people with a disability according to their disability type and life cycle.

The practice and maintenance of self-management behaviors for health promotion are influenced by personal factors such as knowledge and coping skills and environmental factors such as social support and access to resources. Thus, a community-based health promotion system that reflects personal and social factors is a key element of self-management for health promotion [10]. This study aimed to overcome the limitations of previous studies and conduct differentiated research by applying the PRECEDE model, which includes all factors (rather than only one), such as social, epidemiological, behavioral, environmental, and ecological factors.

Various studies have applied the PRECEDE model to investigate the health promotion needs of participants. This model is based on a theory constructed through the complex application of various psychological, social, and physical activity-related theories for the development and evaluation of programs aimed at behavioral changes related to personal health [11]. The PRECEDE model encompasses the pre-diagnosis stage necessary for health promotion in the PRECEDE-PROCEED model, which develops a program through pre-diagnosis and verifies its effectiveness. In the PRECEDE model, interventions are constructed in four steps through a group’s social, epidemiological, behavioral, educational, and ecological diagnoses. Specifically, first, the participant’s social diagnosis and quality of life are determined. Second, epidemiological diagnosis and health conditions are ascertained. Third, behavioral diagnosis and factors related to health promotion behaviors are determined. Finally, the stage of ecological diagnosis explored the influence of determinants derived from epidemiological diagnosis by examining burnout, reinforcing, and enabling factors [12].

Diagnostic research involving the PRECEDE model has been applied in various ways to develop health-related programs [13,14,15], including overseas studies that aim to identify the health characteristics of people with disability [16,17,18]. The necessity of research to identify the characteristics of people with disability is emphasized [16]. Further, the characteristics of people with disability in South Korea are considered different from those of foreign countries, both in terms of environmental and personal factors. Identifying the health characteristics of people with physical disability in South Korea by applying the PRECEDE model can help build a health promotion system in South Korea to increase the satisfaction of people with a physical disability.

Therefore, this study aimed to pre-diagnose the factors affecting the health promotion of people with disability through social, epidemiological, behavioral, and ecological diagnoses by applying the PRECEDE model. The study was intended to identify the characteristics of factors affecting the health promotion of people with physical disability and provide basic data for establishing a health promotion system for people with physical disability in the future. Thus, the research hypotheses were as follows:

**Hypothesis** **1.** *There will be differences in health promotion factors according to the demographic and sociological characteristics of individuals with physical handicaps.*

**Hypothesis** **2.** *There will be differences in health promotion factors according to the disability-related characteristics of individuals with physical handicaps.*

## 2. Materials and Methods

### 2.1. Participants

In this study, participants were selected from those with a physical disability who were registered as of 2022 and who were attending a welfare center for people with disability. Samples were extracted from five regions (Seoul, Gyeonggi-do, Chungcheong-do, Jeolla-do, and Gyeongsang-do) using a systematic stratified cluster sampling method. A total of 1200 people with physical disability were selected as participants, and 200 people with inconsistent responses or non-responses were excluded. Thus, the data of 1000 people (640 males and 360 females) were used for the final analysis. The participants could be grouped into three based on their age ranges: Group A (20–39 or younger; youth); Group B (40–59; middle-aged); Group C (60 or older; old age). In addition, the group comprising individuals aged 60 and older was limited to participants with an intellectual level capable of answering questions on their own. Table 1 shows the participants’ sociodemographic characteristics.

### 2.2. Scales

This study used a structured questionnaire. A scale suitable for the four diagnostic factors of the PRECEDE model was selected for the investigation after discussion with an expert group. The group comprised the following: three people with a physical disability (who each either had sports-related occupations or were employees or members of related organizations); one professor specializing in physical education; two doctors specializing in physical education; one professor specializing in public health; two doctors specializing in public health. First, the quality of life of people with a physical disability was selected for social diagnosis, and the health status of people with a physical disability was selected for epidemiological diagnosis. Further, for behavioral diagnosis, the level of health-promotion behavior of people with a physical disability was identified. For the ecological diagnosis, physical self-efficacy was selected as the predisposing factor, social support as the reinforcing factor, and health promotion behavior intention as the enabling factor. 

#### 2.2.1. Social Diagnosis: Quality of Life

The quality-of-life scale for people with a physical disability, developed by Oh [19] and used by Nam [20] and Shin [21], was modified and supplemented to meet the purpose of this study. This scale comprises 16 items under four sub-factors: physical and mental health, leisure activities, self-identity, general life, and friends and interpersonal relations. All items were scored on a five-point Likert scale. As a result of the exploratory factor analysis, two items in physical and mental health with factor loadings of less than 0.50 were removed [22]. The reliability of the scale expressed as Cronbach’s α, was 0.775 for physical and mental health, 0.871 for leisure activities, 0.899 for self-identity and general life, and 0.886 for friends and interpersonal relations.

#### 2.2.2. Epidemiological Diagnosis: Health Status

The Korean version of the Total Health Index (THI) developed by Lim [23] and the health status items used by Hwang [24] for people with physical disability were modified and supplemented for this study. Considering that the THI developed by Lim [23] comprises items on health with negative meanings and used with negative meanings in previous studies conducted thus far, the items were reverse coded to avoid confusion. The health status scale comprises 16 items in four sub-factors: physical, mental, spiritual, and social health. All items were scored on a five-point Likert scale. As a result of the exploratory factor analysis, items with factor loadings of less than 0.50 were removed from the physical health (one item), mental health (one item), and social health (two items) factors. The reliability of the scale expressed as Cronbach’s α, was 0.762 for physical health, 0.782 for mental health, 0.834 for spiritual health, and 0.672 for social health.

#### 2.2.3. Behavioral Diagnosis: Health Promotion Behavior

The health promotion behavior scale developed by Walker, Sechrist, and Pender [25], translated and revised by Seo [26], and used by Kim [27] for people with a physical disability was modified and supplemented for this study. This scale comprises 12 items under three sub-factors: healthcare, interpersonal relations, and physical activity. All items were scored on a five-point Likert scale. As a result of the exploratory factor analysis, the factor loading was over 0.50 for healthcare, interpersonal relations, and physical activity. The reliability of the scale expressed as Cronbach’s α was 0.683 for healthcare, 0.830 for interpersonal relationships, and 0.885 for physical activity.

#### 2.2.4. Educational and Ecological Diagnosis: Physical Self-Efficacy, Social Support, and Health Behavior Intention

The scales for educational and ecological diagnosis were divided into three main categories: predisposing, reinforcing, and enabling factors. First, for the predisposing factor of physical self-efficacy, the items of the physical self-efficacy scale developed by Ryckman et al. [28] and used by Lee [29] for people with physical disability were modified and supplemented for this study. The scale comprised eight items in two subfactors: physical ability and physical self-expression. As a result of the exploratory factor analysis, one item in physical self-expression with a factor loading of less than 0.50 was removed. The reliability of the scale expressed as Cronbach’s α was 0.777 for physical ability and 0.665 for physical self-expression. Second, for social support, a reinforcing factor, the social support scale developed by Park [30] and used by Lee [31] and Park [32] and the scale used by Oh [33] for people with physical disability were modified and supplemented for this study. The scale comprises eight items under four sub-factors: material, informational, emotional, and appraisal support. As a result of the exploratory factor analysis, the factor loadings were over 0.50 for all items. The reliability of the scale expressed as Cronbach’s α was 0.869 for material support, 0.889 for informational support, 0.890 for emotional support, and 0.886 for appraisal support. Third, for health behavior intention, an enabling factor, the health behavior intention items used by Kim [34] and Lee [35] were modified and supplemented for this study. The health behavior intention scale comprised four items in a single factor. As a result of the exploratory factor analysis, the factor loadings were over 0.50 for all items. The reliability of the scale expressed as Cronbach’s α was 0.849. All items were scored on a five-point Likert scale.

### 2.3. Survey Procedure

To collect data, the authors and research assistants conducted a survey of 1200 people with disability who agreed to participate in the study from 20 May to 30 July 2022. The authors visited welfare centers for people with disability in five districts and obtained prior consent after fully explaining the survey to the person in charge of the institution. After explaining the aim and purpose of the study to people with a physical disability at the welfare center, the questionnaires were distributed to those who voluntarily agreed to participate in the survey. The self-administered method, in which the respondents fill out the questionnaire themselves, was used, and the questionnaires were collected on the spot.

### 2.4. Data Processing

Responses with all items marked as one item among the collected data or with missing items were excluded, and the remaining responses were used for verification with SPSS 22.0. Prior to verifying the research hypothesis, a frequency analysis was performed to identify the characteristics of the participants, followed by an exploratory factor analysis and a Cronbach’s α test to ensure the validity and reliability of the scale. Subsequently, an independent *t*-test and one-way ANOVA were performed as data processing methods to verify the research hypothesis, and Scheffe’s test was used as a post hoc test.

## 3. Results

### 3.1. Differences in Factors Affecting Health Promotion According to Sociodemographic Variables

#### 3.1.1. Differences in Factors Affecting Health Promotion According to Gender

Table 2 shows the results of the independent *t*-test on differences in factors affecting health promotion according to the gender of people with a physical disability. There were partial differences in social (quality of life), epidemiological (health status), behavioral (health promotion behavior), and ecological (physical self-efficacy, social support, and health promotion behavior intention) diagnostic factors according to the participants’ gender. Specifically, the differences were as follows. First, the scores for the physical and mental health of the quality-of-life factor for social diagnosis (t = 2.108, *p* < 0.05) were higher in female participants than in male participants. Second, the mental health score of the health status factor for epidemiological diagnosis (t = 2.665, *p* < 0.01) was higher in male than in female participants. Third, the scores for health care (t = 1.990, *p* < 0.05) and physical activity (t = 2.337, *p* < 0.05) of the health status factor for behavioral diagnosis were higher in male than in female participants. Fourth, the scores for physical ability (t = 4.472, *p* < 0.001), physical activity (t = 5.661, *p* < 0.001), and behavioral intention (t = 2.941, *p* < 0.001) of the physical self-efficacy factor for ecological diagnosis were higher in male than in female participants.

#### 3.1.2. Differences in Factors Affecting Health Promotion According to Age

Table 3 shows the results of the one-way ANOVA on the differences in the factors affecting health promotion according to the age of people with a physical disability. There were partial differences in social (quality of life), epidemiological (health status), and ecological (social support and health promotion behavior intention) diagnostic factors according to gender. Specifically, the differences were as follows. First, among the sub-factors of quality of life for social diagnosis, the scores for physical and mental health (F = 8.076, *p* < 0.001), leisure activity (F = 4.859, *p* < 0.05), and general life (F = 4.863, *p* < 0.01) were higher in Group A than in Groups B and C. Second, the score for the spiritual health of the health status factor for epidemiological diagnosis (t = 3.420, *p* < 0.05) was higher in Group B than in Group C. Third, among the sub-factors of social support for ecological diagnosis, the score for informational support (F = 3.431, *p* < 0.05) was higher in Group C than in Group B, and that for appraisal support (F = 4.815, *p* < 0.01) was higher in Groups A and B than in Group C. The score for the health promotion behavior intention factor for ecological diagnosis (F = 4.354, *p* < 0.05) was higher in Group B than in Group A.

#### 3.1.3. Differences in Factors Affecting Health Promotion According to Income Level

Table 4 shows the results of the one-way ANOVA on the differences in the factors affecting health promotion according to the income level of people with a physical disability. There were partial differences in social (quality of life), epidemiological (health status), behavioral (health promotion behavior), and ecological (social support and health promotion behavior intention) diagnostic factors according to gender. Specifically, the differences were as follows. First, among the sub-factors of quality of life for social diagnosis, the score for physical and mental health (F = 4.893, *p* < 0.01) was higher in Groups A and C than in Group B, whereas the scores for leisure activity (F = 13.022, *p* < 0.001), general life (F = 15.136, *p* < 0.001), and interpersonal relations (F = 3.734, *p* < 0.05) were the highest in Group C, followed by Groups A and C. Second, among the sub-factors of health status for epidemiological diagnosis, the scores for spiritual health (F = 8.889, *p* < 0.001) and physical health (F = 9.364, *p* < 0.001) were the highest in Group C, followed by Groups A and B. However, the score for mental health (F = 4.667, *p* < 0.01) was higher in Group C than in Group B. Third, among the sub-factors of health promotion behavior for behavioral diagnosis, the score for healthcare (F = 3.864, *p* < 0.05) was higher in Group C than in Groups A and B, whereas those for interpersonal relations (F = 9.071, *p* < 0.001) and physical activity (F = 13.943, *p* < 0.001) were higher in Group C than in Groups B and A. Fourth, among the sub-factors of physical self-efficacy for ecological diagnosis, the score for physical ability (F = 9.133, *p* < 0.001) was higher in Group C than in Groups B and A, and that for physical expression (F = 8.192, *p* < 0.001) was higher in Group C than in Groups A and B. Among the sub-factors of social support, the score for informational support (F = 3.992, *p* < 0.05) was higher in Group C than in Groups A and B, and that for appraisal support (F = 7.487, *p* < 0.001) was higher in Group C than in Groups A and B. The score for the health promotion behavior intention factor (F = 18.515, *p* < 0.05) was higher in Group C than in Groups A and B.

### 3.2. Differences in Factors Affecting Health Promotion According to Disability-Related Variables

#### 3.2.1. Differences in Factors Affecting Health Promotion According to Time of Onset

Table 5 shows the results of the independent *t*-test on the differences in the factors affecting health promotion according to the time of onset for people with a physical disability. Partial differences were observed in social (quality of life), epidemiological (health status), behavioral (health promotion behavior), and ecological (physical self-efficacy and health promotion behavior intention) diagnostic factors according to the time of onset. Specifically, the differences were as follows. First, the scores for all sub-factors of quality of life for social diagnosis, including physical and mental health (t = 2.817, *p* < 0.01), leisure activity (t = 3.344, *p* < 0.001), general life (t = 2.682, *p* < 0.01), and interpersonal relations (t = 2.156, *p* < 0.05), were higher in the congenital disability group than in the acquired disability group. Second, among the sub-factors of quality of life for epidemiological diagnosis, the scores for spiritual health (t = 3.954, *p* < 0.001), physical health (t = 2.447, *p* < 0.05), and social health (t = 3.409, *p* < 0.001) were higher in the congenital disability group than in the acquired disability group. Third, the score for interpersonal relationships (t = 2.602, *p* < 0.05) of the health promotion behavior factor for behavioral diagnosis was higher in the congenital disability group than in the acquired disability group. Fourth, the scores for physical expression (t = −2.496, *p* < 0.05) and behavioral intention (t = −2.134, *p* < 0.05) of the physical self-efficacy factor for ecological diagnosis were higher in the congenital disability group than in the acquired disability group.

#### 3.2.2. Differences in Factors Affecting Health Promotion According to Degree of Disability

Table 6 shows the results of the independent *t*-test on the differences in the factors affecting health promotion according to the degree of disability. Partial differences were observed in social (quality of life), epidemiological (health status), behavioral (health promotion behavior), and ecological (physical self-efficacy and health promotion behavior intention) diagnostic factors according to the degree of disability. Specifically, the differences were as follows. First, among the sub-factors of quality of life, the scores for physical and mental health (t = 4.508, *p* < 0.001) and general life (t = 3.381, *p* < 0.01) were higher in the severe disability group than in the mild disability group. Second, among the sub-factors of health status for epidemiological diagnosis, the scores for spiritual health (t = 2.268, *p* < 0.05), physical health (t = 3.987, *p* < 0.001), and social health (t = 2.616, *p* < 0.01) were higher in the severe disability group than in the mild disability group. Third, the score for interpersonal relationships (t = 3.002, *p* < 0.05) of the health promotion behavior factor for behavioral diagnosis was higher in the severe disability group than in the mild disability group. Fourth, among the sub-factors of physical self-efficacy for ecological diagnosis, the scores for physical expression (t = 2.438, *p* < 0.05) and behavioral intention (t = 2.907, *p* < 0.01) were higher in the congenital disability group than in the acquired disability group.

## 4. Discussion

### 4.1. Verification of Differences in Factors Affecting Health Promotion According to Personal Background Variables

To test Hypothesis 1, the differences in the factors affecting health promotion according to personal background variables were examined, which yielded the following results:

Differences emerged in the factors influencing health promotion among people with a physical disability according to the sociodemographic variables of gender, age, and income level. Consequently, partial differences were found in the quality of life, health status, health promotion behavior, physical self-efficacy, social support, and health behavior intention.

First, there were differences according to gender, age, and income level in the social diagnostic factor of quality of life. Verifying the differences according to gender showed that scores for physical and mental health were higher in male than in female participants. Verifying the differences according to age showed that the scores for physical and mental health, leisure activities, self-identity, and general life were higher in participants aged 30 and younger. Further, verifying the differences according to income level showed that the scores for physical and mental health, leisure activities, self-identity, general life, and interpersonal relationships were higher in the group earning KRW 2.01 million or more per month. These results were partially consistent with those of previous studies on the quality of life of people with physical disability [19,36,37,38]. The results of this study showed a higher quality of life in male than in female participants, consistent with Hwang [38]. In South Korean society, men engage in more diverse social activities than women, and barriers to entry are considered lower for men than for women, especially because many club cultures, such as leisure sports clubs, are centered on men.

This study’s results also demonstrated a higher quality of life in younger participants, which was consistent with the study of Kim [36], who reported that younger age groups engaged in more group activities at school or work and their ability to collect various details through cell phones and the internet improved their quality of life. In other words, the quality of life of people with a physical disability can be improved through group activities and the collection of various types of information. In particular, considering this study’s finding that those aged 39 or younger had high levels of self-identity and general quality of life, these factors seemed to be improved through social activities. However, Oh [19] reported that quality of life was higher in older groups. Nevertheless, in that study, participants were limited to those engaged in leisure sports activities, which had a higher participation rate for those in their 50s or older. This may have contributed to the statistically significant high levels of physical and mental health in the groups in their 50s or older. Therefore, the results were different from those of this study, which reported high levels of self-identity and general quality of life, the sub-factors of quality of life, in those aged 39 or younger.

Another result from this study demonstrated a higher quality of life in the higher-income group, consistent with Park [37], Oh [19], and Hwang [38]. Park [37] reported that people with a physical disability could have various independent experiences with a certain level of economic power guaranteed to enhance their quality of life. In other words, it appears that people with physical disability incur various additional costs in moving or participating in new activities compared to those without disability. Consequently, a certain level of economic power helps to keep their activities from being restricted. Therefore, efforts to improve the quality of life of people with physical disability in their 40s or older with a monthly income of KRW 1.5 million or less are necessary.

This study also showed partial differences according to gender, age, and income level in health status, which is an epidemiological diagnostic factor. When verifying the differences according to gender, the results showed that mental health scores were higher in males than in females. Verifying the differences according to age showed that the score for spiritual health was higher in participants in their 40–50 s. Verifying the differences according to income level showed that the scores for mental health, physical health, and spiritual health were higher in the group earning KRW 1.01 million to 1.5 million per month. These results were partially consistent with previous studies on the health status of people with disability [39,40]. Jeong [40] reported that female participants were less socially active than their male counterparts and that they experienced a sense of alienation and disgrace that negatively affected their mental health. In particular, considering that it is still considerably difficult for women with disability to find employment in South Korea, the mental health of women with a disability was lower than that of men with disability. This was because the former lacked social activities and experienced a sense of loss and alienation. This result was consistent with those reported by Park [39] and Jeong [40]. Jeong [40] reported that older adults perceived their deteriorating health condition as aging. Nonetheless, there were restrictions on periodic health care in old age and at the time of economic retirement. The results of this study indicating a higher level of health status given a higher income level contradicted the results of Jeong [40]. Jeong [40] suggested that to increase the level of income in old age, continuous economic activity is required. Nevertheless, those engaged in continuous economic activity in old age neglected their health, despite their high-income level, while investing in their children rather than themselves. However, for people with a physical disability to have a high income, they must be able to maintain an income of KRW 1.51 million or more if they are beneficiaries of industrial accident insurance or have a job. In particular, most of the groups with an income of KRW 1.01 million to 1.5 million were expected to be basic livelihood security recipients. Accordingly, various economic difficulties were judged to contribute to poor health status.

Third, there were differences in health promotion behavior, which is a behavioral diagnostic factor, according to gender and income level. When verifying the differences according to gender, the score for physical activity was higher in male than in female participants. When verifying the differences according to income level, the scores for healthcare, interpersonal relations, and physical activity were higher in the group earning KRW 1.51 million or more. The results of this study were consistent with previous studies on health-promotion behavior [19]. Oh [19] reported that men with disability are more physically active than women with disability. People with a physical disability who had a higher income level showed higher levels of health care, interpersonal relations, and physical activity, supporting the results of this study. While men with disability stayed physically active through social and club activities, women faced many limitations due to a lack of social experience and the lack of clubs for women compared to men. As the health promotion activities of people with physical disability incur an incidental cost compared to those of people without disability, more varied health promotion activities seem to be available with a higher income level.

Fourth, there were differences according to gender and income level in physical self-efficacy, an educational and ecological diagnostic factor, age and income level in social support, as well as gender, age, and income level in health promotion behavior intention. Specifically, as a result of verifying the differences according to gender, the score for physical self-efficacy was higher in male than in female participants. When verifying the differences according to income level, the scores for physical income and expression were higher in the group earning KRW 1.51 million or higher per month. Regarding social support, informational support and appraisal support were higher in the group aged in their 40s to 50s and in the group earning KRW 1.51 million or higher per month. Health behavior intention was higher in men, in the group in their 40s to 50s, and in the group earning KRW 1.51 million or higher per month. Owing to the scarcity of studies that verified the difference in gender and income level related to physical self-efficacy, health behavioral intention, and social support for people with a physical disability, there are insufficient previous studies to support the results of this study. Nevertheless, the present findings, which demonstrate differences in physical self-efficacy, health behavior intention, and social support according to gender and income level, suggest that these factors are highly significant for people with disability. In particular, women had lower physical self-efficacy and health-promoting behavioral intentions than men, and those with a higher income level had higher physical self-efficacy, health behavior intention, and social support. This suggests that women and people with disability with low-income levels are vulnerable in terms of health promotion.

Considered together, in the social diagnosis (quality of life), the older and low-income groups are considered vulnerable; in the epidemiological diagnosis (health status), the older and high-income groups are considered vulnerable; and in the behavioral diagnosis (health promotion behavior), the high-income group is considered vulnerable. In ecological diagnosis (physical self-efficacy, health behavior intention, and social support), women and low-income groups are considered vulnerable. In other words, a health promotion system should be established to improve the quality of life of groups that are older and have lower income. Meanwhile, the older and high-income groups, who had a higher health status, thought negatively about their health, suggesting that it is difficult to assume that the high-income group had an advantage in health promotion. Considering that the income level of people with a physical disability was somewhat lower than that of people without disability, even the high-income group’s level of income seemed insufficient for old age. Furthermore, as women and low-income groups are considered vulnerable in ecological diagnosis, it seems necessary to develop a program to encourage their participation.

### 4.2. Differences in Factors Affecting Health Promotion According to Disability-Related Variables

To test Hypothesis 2, the differences in the factors affecting health promotion according to disability-related variables were examined.

As a result of verifying the differences in factors influencing health promotion according to the time of onset and degree of disability as the disability-related variables, partial differences were found in the quality of life, health status, health promotion behavior, physical self-efficacy, and health behavior intention. Specifically, they were as follows:

First, there were differences according to the time of onset and degree of disability in the quality of life, which is a social diagnostic factor. As a result of verifying the differences according to the time of onset, the scores for physical and mental health, leisure activities, self-identity, general life, and interpersonal relations were higher in people with a congenital physical disability. According to the degree of disability, the scores for physical and mental health, self-identity, and general life were higher among people with severe disability. These results are partially consistent with those of previous studies on the quality of life of people with a physical disability according to the time of onset and degree of disability [20,36,37,38]. The results showing a higher quality of life in people with congenital physical disability than in people with acquired physical disability were consistent with the study of Kim [36], who reported that people with congenital disability were more realistic and psychologically stable than those with acquired disability, thereby having a higher quality of life. In other words, people with acquired disability have lower self-esteem compared to their state before they acquired a disability. They had considerable difficulty accepting their present life due to memories of the past, thereby experiencing a lower quality of life. While few studies have verified the differences in the quality of life of people with a physical disability, this study has shown a sizeable difference based on the time of disability onset.

The finding of a higher quality of life in people with severe disability was partially consistent with the findings of Kim [36], Nam [20], Park [37], and Hwang [38]. Hwang [38] reported that people with severe disability adapted to their disability for longer, with well-systematized physical activities for those with severe disability providing various experiences. In other words, people’s degree of disability directly affects their range of activities. More diverse programs are available for people with severe disability, thereby contributing to an increase in their quality of life.

Second, there were differences in health status according to the time of onset and degree of disability, which is an epidemiological diagnostic factor. An examination of the differences according to the time of onset revealed that the scores for physical, mental, and spiritual health were higher in people with a congenital disability. Regarding the degree of disability, the scores for physical, mental, and spiritual health were higher in people with severe disability. These results were partially consistent with those of previous studies on health status according to the time of onset and degree of disability [39,41]. The results of this study, which indicate a higher health status perceived by people with acquired disability, suggest that they were aware of the rapid deterioration in their health status due to their past experience of living without disability. This supports the results of Park [38], who reported that the sudden onset of disability caused participants to experience considerable difficulty in managing their health compared to before they acquired a disability. Those with acquired disability seemed to have more difficulty managing their health due to their smaller range of possible activities and increased personal physical limitations. On the other hand, the results of this study, which show higher scores for physical, mental, and spiritual health in people with severe disability, were consistent with those of Im [41], who reported that a more systematic welfare system was provided to people with severe physical disability than those with a mild physical disability. This may have contributed to people with a mild physical disability being more vulnerable in terms of healthcare. In other words, people with a physical disability who were determined to be “mild” based on the existing welfare policy’s classifications but who still engaged in limited activities were likely to have healthcare difficulties.

Third, there were differences in health promotion behavior, which is a behavioral diagnostic factor, according to the time of onset and degree of disability. An examination of the differences according to the time of onset showed that the score for interpersonal relations was higher in people with a congenital disability, and the score for physical activity was higher in people with severe disability. These results are consistent with those of Kim [42], who reported that people with congenital disability showed higher health promotion behavior than those with acquired disability. Therefore, these prior results are consistent with this study’s findings that people with congenital disability have more diverse experiences in health management than those with an acquired disability because they have received education on various health promotion behaviors from their parents or schools as they grew up.

Fourth, there were differences according to time of onset and degree of disability in physical self-efficacy—an educational and ecological diagnostic factor—and according to time of onset and degree of disability in health promotion behavior intention. There were no differences in social support. Regarding physical self-efficacy, the score for physical expression was higher among people with acquired disability. As a result of examining the differences according to the degree of disability, the score of physical expression was higher in people with severe disability. Moreover, health behavioral intentions were higher among people with severe disability. Due to the scarcity of studies on physical self-efficacy, health behavioral intention, and social support for people with physical disability verifying the difference in the time of onset and degree of disability, there were insufficient studies to support the present results. Nevertheless, the results of this study showed differences in physical self-efficacy, health behavior intention, and social support according to time of onset and degree of disability, suggesting that the time of onset and degree of disability for people with disability were highly significant factors. The high physical self-efficacy in people with acquired disability, low physical self-efficacy, and health promotion behavior intention in people with mild disability suggest that those with congenital or severe disability are vulnerable to encountering healthcare difficulties.

Considered together, in the social diagnosis (quality of life), people with congenital or severe disability are considered vulnerable; in the epidemiological diagnosis (health status), those with acquired or mild disability are considered vulnerable; and in the behavioral diagnosis (health promotion behavior), those with acquired disability are considered vulnerable. In other words, the social, epidemiological, and behavioral diagnoses that identify people with acquired or mild disability as vulnerable groups suggest a need for health promotion programs for those with acquired or mild disability. In ecological diagnosis (physical self-efficacy, health behavior intention, and social support), people with congenital or mild disability are considered vulnerable. In other words, the ecological diagnosis that determined the possibility of health promotion behavior identified people with congenital or mild disability as vulnerable groups, suggesting a need for health promotion programs that encourage their participation.

Therefore, since acquired and mildly handicapped people are more vulnerable to health complications, targeted health promotion programs should be developed for them.

## 5. Conclusions

This study aimed to analyze the factors affecting the health of people with physical disability through social, epidemiological, behavioral, and ecological diagnoses by partially applying the PRECEDE model. This study is considered to have overcome the limitations of previous studies that were conducted without considering the characteristics of people with disability. The following conclusions were drawn from this study’s findings.

First, in the social diagnosis (quality of life), the older and low-income groups were considered vulnerable; in the epidemiological diagnosis (health status), the older and high-income groups were considered vulnerable groups; and in the behavioral diagnosis (health promotion behavior), the high-income group was considered vulnerable. In ecological diagnosis (physical self-efficacy, health behavior intention, and social support), women and low-income groups were considered vulnerable. Second, in the social diagnosis (quality of life), people with congenital or severe disability were considered vulnerable groups; in the epidemiological diagnosis (health status), those with acquired or mild disability are considered vulnerable groups; and in the behavioral diagnosis (health promotion behavior), those with acquired disability are considered vulnerable groups.

The following suggestions are made for follow-up research by changing the perspectives and methods to address the limitations of this study. First, this study had a limitation in that the characteristics of other types of disability were not considered, as the participants of this study were limited to those with a physical disability. Therefore, it will be necessary to identify the health characteristics of various types of disability other than a physical disability and analyze their health promotion needs. Second, this study aimed to determine the health promotion characteristics of people with a physical disability. Therefore, based on these results, continuous follow-up studies will be required to establish a health promotion system for people with a physical disability.

## Figures and Tables

**Table 1:** Sociodemographic characteristics of the subjects.

Characteristic		Division	Pilot Survey	
			Frequency (People)	Ratio (%)
Personal background variables	Gender	Male	640	64.0
		Female	360	36.0
	Age	39 or younger (Group A)	290	29.0
		40–59 (Group B)	165	16.5
		60 or older (Group C)	545	54.5
	Income level	Less than KRW 1 million	640	64.0
		KRW 1.01 million to 1.5 million	247	24.7
		KRW 1.51 million or more	113	11.3
Disability-related variables	Disability type	Congenital	339	33.9
		Acquired	661	66.1
	Degree of disability	Severe disability	820	82
		Mild disability	180	18
Total			1000	100.0

**Table 2:** Verification of differences in factors affecting health promotion according to gender.

Division			*n*	Mean	Standard Deviation	*t*
Quality of life	Physical and mental	Male	640	3.4000	1.02557	2.108 *
		Female	360	3.2542	1.09187	
	Leisure activity	Male	640	3.5324	0.99521	1.780
		Female	360	3.4153	1.00527	
	General life	Male	640	3.4909	0.97426	1.409
		Female	360	3.3979	1.04736	
	Interpersonal relation	Male	640	3.5203	0.98153	0.908
		Female	360	3.4618	0.97216	
Health status	Spiritual health	Male	640	2.3207	0.95915	−0.863
		Female	360	2.3764	1.01314	
	Physical health	Male	640	2.4073	1.03885	−0.907
		Female	360	2.4694	1.04183	
	Mental health	Male	640	2.3359	0.92408	2.665 **
		Female	360	2.5028	0.99488	
	Social health	Male	640	2.7836	1.07087	−0.742
		Female	360	2.8361	1.08205	
Health promotion behavior	Healthcare	Male	640	3.2605	0.84173	1.990 *
		Female	360	3.1493	0.86064	
	Interpersonal relation behavior	Male	640	3.5242	0.90166	0.828
		Female	360	3.4750	0.90403	
	Physical activity	Male	640	3.4438	1.06432	2.337 *
		Female	360	3.2799	1.06507	
Physical self-efficacy	Physical ability	Male	640	2.9547	0.92029	4.472 ***
		Female	360	2.6910	0.84825	
	Physical expression	Male	640	3.3245	0.89148	5.661 ***
		Female	360	2.9889	0.91441	
Social support	Material support	Male	640	3.4719	0.93906	−0.283
		Female	360	3.4894	0.93637	
	Informational support	Male	640	3.5059	0.97417	1.675
		Female	360	3.4007	0.91359	
	Mental support	Male	640	3.5270	0.93894	0.590
		Female	358	3.4902	0.94874	
	Appraisal support	Male	640	3.5902	0.93592	1.281
		Female	358	3.5119	0.91001	
Behavioral intention	Behavioral intention	Male	640	3.7188	0.99579	2.941 **
		Female	360	3.5250	1.00715	

* *p* < 0.05, ** *p* < 0.01, *** *p* < 0.001.

**Table 3:** Verification of the differences in factors affecting health promotion according to age.

Division			*n*	Mean	Standard Deviation	*t*/*F*	Post-Hoc Test
Quality of life	Physical and mental	A	290	3.555	1.106	8.076 ***	C,B < A
		B	165	3.264	1.019		
		C	545	3.262	1.018		
	Leisure activity	A	290	3.634	0.935	4.586 *	B,C < A
		B	165	3.374	1.009		
		C	545	3.449	1.024		
	General life	A	290	3.611	1.001	4.863 **	C,B < A
		B	165	3.405	0.948		
		C	545	3.392	1.011		
	Interpersonal relation	A	290	3.545	0.971	1.083	
		B	165	3.556	0.950		
		C	545	3.458	0.990		
Health status	Spiritual health	A	290	3.734	1.008	3.420 *	C < B
		B	165	3.770	0.927		
		C	545	3.586	0.974		
	Physical health	A	290	3.477	1.168	1.919	
		B	165	3.661	1.005		
		C	545	3.593	0.974		
	Mental health	A	290	3.568	0.986	2.020	
		B	165	3.739	0.794		
		C	545	3.582	0.977		
	Social health	A	290	3.236	1.215	0.844	
		B	165	3.261	0.962		
		C	545	3.158	1.027		
Health promotion behavior	Healthcare	A	290	3.291	0.877	2.011	
		B	165	3.129	0.771		
		C	545	3.211	0.856		
	Interpersonal relation behavior	A	290	3.571	0.945	1.921	
		B	165	3.562	0.831		
		C	545	3.456	0.898		
	Physical activity	A	290	3.354	1.059	0.166	
		B	165	3.395	0.935		
		C	545	3.398	1.109		
Physical self-efficacy	Physical ability	A	290	2.947	0.974	1.976	
		B	165	2.805	0.833		
		C	545	2.830	0.883		
	Physical expression	A	290	3.205	0.973	0.006	
		B	165	3.210	0.822		
		C	545	3.201	0.909		
Social support	Material support	A	290	3.540	0.968	2.031	
		B	165	3.550	0.984		
		C	545	3.424	0.905		
	Informational support	A	290	3.530	0.975	3.431 *	C < B
		B	165	3.589	0.930		
		C	545	3.398	0.945		
	Mental support	A	290	3.569	0.979	0.949	
		B	165	3.536	0.977		
		C	543	3.477	0.911		
	Appraisal support	A	290	3.640	0.910	4.815 **	C < A,B
		B	165	3.694	0.945		
		C	543	3.481	0.924		
Behavioral intention	Behavioral intention	A	290	3.527	0.984	4.354 *	A < B
		B	165	3.809	0.976		
		C	545	3.666	1.016		

* *p* < 0.05, ** *p* < 0.01, and *** *p* < 0.001; note: A = 20–39 or younger; B = 40–59; C = 60 or older.

**Table 4:** Verification of differences in factors affecting health promotion according to income level.

Division			*n*	Mean	Standard Deviation	*t*/*F*	Post-Hoc Test
Quality of life	Physical and mental	A	640	3.4000	1.08598	4.893 **	B < A,C
		B	247	3.1680	1.00158		
		C	113	3.4425	0.91552		
	Leisure activity	A	640	3.4996	1.02373	13.002 ***	B < A < C
		B	247	3.2945	0.95266		
		C	113	3.8650	0.84781		
	General life	A	640	3.4773	1.02725	15.163 ***	B < A < C
		B	247	3.2304	0.95464		
		C	113	3.8407	0.81426		
	Interpersonal relation	A	640	3.5086	1.02260	3.734 *	B,A < C
		B	247	3.3887	0.89474		
		C	113	3.6881	0.86314		
Health status	Spiritual health	A	640	3.695	1.001	8.889 ***	B < A < C
		B	247	3.461	0.892		
		C	113	3.894	0.966		
	Physical health	A	640	3.608	1.039	9.364 ***	B < A < C
		B	247	3.355	0.976		
		C	113	3.829	1.101		
	Mental health	A	640	3.635	0.970	4.667 **	B < C
		B	247	3.456	0.904		
		C	113	3.749	0.929		
	Social health	A	640	3.259	1.131	2.884	
		B	247	3.089	0.982		
		C	113	3.088	0.907		
Health promotion behavior	Healthcare	A	640	3.1934	0.87580	3.864 *	A,B < C
		B	247	3.1953	0.82612		
		C	113	3.4292	0.71921		
	Interpersonal relation behavior	A	640	3.4789	0.95094	9.071 ***	B,A < C
		B	247	3.4261	0.81079		
		C	113	3.8385	0.72804		
	Physical activity	A	640	3.3691	1.09958	13.943 ***	B,A < C
		B	247	3.2156	0.99991		
		C	113	3.8429	0.88527		
Physical self-efficacy	Physical ability	A	640	2.8172	0.93906	9.133 ***	B,A < C
		B	247	2.8148	0.79783		
		C	113	3.1991	0.84921		
	Physical expression	A	640	3.1349	0.93820	8.192 ***	A,B < C
		B	247	3.2456	0.85695		
		C	113	3.5015	0.83110		
Social support	Material support	A	640	3.4823	0.96332	1.680	
		B	247	3.4099	0.84237		
		C	113	3.6040	0.98126		
	Informational support	A	640	3.4613	0.99538	3.992 *	B,A < C
		B	247	3.3846	0.84905		
		C	113	3.6881	0.90110		
	Mental support	A	638	3.5380	0.95833	1.824	
		B	247	3.4180	0.88409		
		C	113	3.5863	0.96580		
	Appraisal support	A	638	3.5341	0.95581	7.487 ***	B,A < C
		B	247	3.4919	0.83765		
		C	113	3.8739	0.89401		
Behavioral intention	Behavioral intention	A	640	3.5715	1.02534	18.515 ***	A,B < C
		B	247	3.6073	0.96200		
		C	113	4.1792	0.79582		

* *p* < 0.05, ** *p* < 0.01, and *** *p* < 0.001; note: A = KRW 1 million or lower, B = KRW from 1.01 million to 1.5 million, and C = KRW 1.51 million or higher.

**Table 5:** Verification of differences in factors affecting health promotion according to time of onset.

Division			*n*	Mean	Standard Deviation	*t*
Quality of life	Physical and mental	Congenital	339	3.478	1.099	2.817 **
		Acquired	661	3.281	1.021	
	Leisure activity	Congenital	339	3.637	1.011	3.344 ***
		Acquired	661	3.415	0.987	
	General life	Congenital	339	3.576	1.065	2.682 **
		Acquired	661	3.397	0.963	
	Interpersonal relation	Congenital	339	3.592	1.005	2.156 *
		Acquired	661	3.452	0.961	
Health status	Spiritual health	Congenital	339	3.829	0.966	3.954 ***
		Acquired	661	3.572	0.974	
	Physical health	Congenital	339	3.682	1.097	2.447 *
		Acquired	661	3.513	1.005	
	Mental health	Congenital	339	3.673	0.984	1.631
		Acquired	661	3.569	0.936	
	Social health	Congenital	339	3.358	1.112	3.409 ***
		Acquired	661	3.115	1.046	
Health promotion behavior	Healthcare	Congenital	339	3.261	0.849	1.081
		Acquired	661	3.200	0.850	
	Interpersonal relation behavior	Congenital	339	3.610	0.946	2.602 **
		Acquired	661	3.453	0.875	
	Physical activity	Congenital	339	3.385	1.063	0.004
		Acquired	661	3.385	1.070	
Physical self-efficacy	Physical ability	Congenital	339	2.833	0.926	−0.680
		Acquired	661	2.874	0.892	
	Physical expression	Congenital	339	3.103	1.020	−2.496 *
		Acquired	661	3.255	0.850	
Social support	Material support	Congenital	339	3.483	1.057	0.118
		Acquired	661	3.476	0.871	
	Informational support	Congenital	339	3.493	1.044	0.602
		Acquired	661	3.455	0.905	
	Mental support	Congenital	339	3.493	1.041	−0.509
		Acquired	659	3.525	0.888	
	Appraisal support	Congenital	339	3.552	1.010	−0.257
		Acquired	659	3.568	0.882	
Behavioral intention	Behavioral intention	Congenital	339	3.555	1.106	−2.134 *
		Acquired	661	3.697	0.944	

* *p* < 0.05, ** *p* < 0.01, and *** *p* < 0.001.

**Table 6:** Verification of differences in factors affecting health promotion according to degree of disability.

Division			*n*	Mean	Standard Deviation	*t*
Quality of life	Physical and mental	Severe disability	820	3.417	1.034	4.508 ***
		Mild disability	180	3.031	1.077	
	Leisure activity	Severe disability	820	3.518	0.996	1.854
		Mild disability	180	3.365	1.012	
	General life	Severe disability	820	3.507	0.999	3.381 ***
		Mild disability	180	3.230	0.985	
	Interpersonal relation	Severe disability	820	3.492	0.993	−0.495
		Mild disability	180	3.532	0.907	
Health status	Spiritual health	Severe disability	820	3.692	0.995	2.268 *
		Mild disability	180	3.510	0.886	
	Physical health	Severe disability	820	3.631	1.036	3.987 ***
		Mild disability	180	3.293	1.017	
	Mental health	Severe disability	820	3.611	0.965	0.465
		Mild disability	180	3.574	0.900	
	Social health	Severe disability	820	3.239	1.099	2.616 **
		Mild disability	180	3.008	0.937	
Health promotion behavior	Healthcare	Severe disability	820	3.227	0.836	0.527
		Mild disability	180	3.190	0.914	
	Interpersonal relation behavior	Severe disability	820	3.505	0.907	−0.144
		Mild disability	180	3.515	0.881	
	Physical activity	Severe disability	820	3.432	1.065	3.002 **
		Mild disability	180	3.169	1.054	
Physical self-efficacy	Physical ability	Severe disability	820	2.882	0.911	1.687
		Mild disability	180	2.757	0.864	
	Physical expression	Severe disability	820	3.237	0.937	2.438 *
		Mild disability	180	3.054	0.782	
Social support	Material support	Severe disability	820	3.494	0.945	1.148
		Mild disability	180	3.406	0.902	
	Informational support	Severe disability	820	3.480	0.955	0.819
		Mild disability	180	3.415	0.950	
	Mental support	Severe disability	818	3.530	0.946	1.178
		Mild disability	180	3.439	0.923	
	Appraisal support	Severe disability	818	3.576	0.938	0.993
		Mild disability	180	3.500	0.876	
Behavioral intention	Behavioral intention	Severe disability	820	3.692	1.003	2.907 **
		Mild disability	180	3.453	0.988	

* *p* < 0.05, ** *p* < 0.01, and *** *p* < 0.001.
